# The impact of patient characteristics on enzalutamide pharmacokinetics and how this relates to treatment toxicity and efficacy in metastatic prostate cancer patients

**DOI:** 10.1007/s00280-020-04039-7

**Published:** 2020-02-19

**Authors:** Guillemette E. Benoist, Inge M. van Oort, David M. Burger, Niven Mehra, Nielka P. van Erp

**Affiliations:** 1 Department of Pharmacy, Radboud University Medical Center, Radboud Institute for Health Sciences, Nijmegen, The Netherlands; 2 Department of Urology, Radboud University Medical Center, Nijmegen, The Netherlands; 3 Department of Medical Oncology, Radboud University Medical Center, Nijmegen, The Netherlands

**Keywords:** Enzalutamide, Fatigue, Pharmacokinetics, Metastatic castration-resistant prostate cancer

## Abstract

**Purpose:**

The aim of the study is to investigate the influence of patient characteristics, age and body mass index (BMI), on pharmacokinetics of enzalutamide, and to study the relationships between drug exposure and enzalutamide efficacy and toxicity, in mCRPC patients.

**Methods:**

Data were collected in a longitudinal cohort study (ANDROPS) and a prospective observational study (ILUMINATE), both in mCRPC patients treated with enzalutamide. To investigate the influence of age and BMI on exposure, enzalutamide and N-desmethylenzalutamide levels were compared by ANOVA. To investigate the relation of exposure versus time to progression (TTP), the sum plasma levels were divided into quartiles and compared by Kaplan–Meier analysis. To assess the relation of exposure with fatigue, plasma levels in patients experiencing fatigue vs. no fatigue were compared by and independent t test.

**Results:**

Data of 68 mCRPC patients were included for analysis. Plasma levels were not different for age or BMI. No difference in TTP between both studies was observed (383 days (95% CI 287–859), and 567 days (95% CI 351–NR), *p* = 0.36). Kaplan–Meier analysis of quartiles of sum levels showed no difference for TTP. Fatigue was reported by 22 patients, no difference in sum plasma levels was observed between patients with and without fatigue.

**Conclusions:**

We observed that age and BMI did not influence systemic exposure in patients treated with enzalutamide. No relation of exposure with efficacy or fatigue was observed. Further research using enzalutamide at a lower dose is needed to understand the relation of enzalutamide exposure and fatigue.

## Introduction

Metastatic castration-resistant prostate cancer (mCRPC) is the second most common cancer in men in western countries [1]. Currently, the median overall survival for mCRPC patients is varying between 19 and 35 months [2, 3]. Prior to 2011, docetaxel was the only therapy available, but during the last decade, multiple life-prolonging therapies became available: e.g. cabazitaxel, radium-223, and two oral anti-androgen directed therapies, abiraterone acetate and enzalutamide [4–7]. Recently, the indication for enzalutamide treatment was broadened from metastatic to non-metastatic CRPC [8, 9]. Furthermore, enzalutamide showed improved overall survival in the hormone-sensitive setting which may broaden the patient population even more in the nearby future [10].

During ageing, several factors that may affect the pharmacokinetics of enzalutamide change, for example an increase in body fat and decrease in clearance of the liver with older age [11]. Furthermore, in the population pharmacokinetic analysis, enzalutamide exposure was ~ 20% lower in mCRPC patients with a higher weight (120 kg vs. 70 kg), this may be attributed to an increased volume of distribution. Finally, it was shown that CRPC patients treated outside of clinical trials had different baseline characteristics compared to study patients, which may be of influence on the pharmacokinetics of enzalutamide and its active metabolite [12].

However, limited data are available on the relation between enzalutamide and N-desmethylenzalutamide exposure and patient characteristics. Only one pharmacokinetic study, outside the registration study, of enzalutamide in relation to patient characteristics is currently available, showing that age did not affect enzalutamide exposure [13]. The population pharmacokinetic analysis did not reveal an exposure response relationship for overall survival also no consistent relation was found for exposure and toxicity.

One of the most commonly reported side effects of enzalutamide is fatigue, occurring in approximately 36% of the patients during the PREVAIL trial [3]. The hypothesis is that fatigue might be caused by the exposure to both enzalutamide and its active metabolite N-desmethylenzalutamide. Both cross the blood–brain barrier and have high affinity for the GABA receptor. This has previously been described in mice and rats for enzalutamide and N-desmethylenzalutamide as well as for other anti-androgens, such as nilutamide and flutamide [9, 14]. While fatigue was a dose-dependent adverse event in the phase I study, no consistent relationship between enzalutamide plasma concentrations and fatigue was found in the pharmacometric analysis [15, 16].

Eliasson et al. showed that patients with advanced prostate cancer prefer a therapy that (1) controls bone pain, (2) delays chemotherapy, (3) avoids side effects such as memory loss and extreme tiredness. This patient preference underlines the significance of understanding the potential relation between systemic exposure to enzalutamide plus N-desmethylenzalutamide and fatigue. This will help to improve and potentially even prevent central nervous system toxicity of this therapy for future patients [17].

In summary, by an improved understanding of the effect of patient characteristics on the pharmacokinetics, and the relationship of exposure with efficacy and toxicity, treatment with enzalutamide might in the future be individualised.

## Materials and methods

For this analysis, data of two studies were used. ANDROPS was a longitudinal observational cohort study in patients treated with 160 mg enzalutamide once daily, in the Radboud university medical center (Nijmegen, the Netherlands). Patients were included from September 2015 to February 2019 while on treatment or starting treatment with enzalutamide.

ILUMINATE was a multi-center prospective study in patients with mCRPC who started treatment with enzalutamide (ClinicalTrials.gov NCT02471469). Patients were included from July 2015 to September 2017 in five hospitals in the Netherlands (Canisius Wilhelmina Hospital, Gelderse Vallei hospital, Francisus gasthuis and Vlietland hospital, Reinier de Graaf Hospital, Radboud university medical center). Patients who did not receive prior chemotherapy and patients who had upfront docetaxel according to CHAARTED/STAMPEDE trial protocols were eligible for this study.

After informed consent, blood was drawn at standard patient visits in the outpatient clinic at random times in the ANDROPS study and at prespecified moments in the ILUMINATE study (pre-dose after 1, 3 and 6 months of enzalutamide treatment). Each blood sample was collected into an ethylenediaminetetraacetic acid containing tube, and for quantification of enzalutamide and N-desmethylenzalutamide concentrations, a validated method as previously described was used [18]. Due to the long half-life of enzalutamide (mean 5.8 days) and its active metabolite (mean 8.6 days), there is a small difference between the maximum and minimum concentrations of enzalutamide and N-desmethylenzalutamide (ratio of 1.25 for enzalutamide), allowing random time of sampling.

**Fig. 1:**
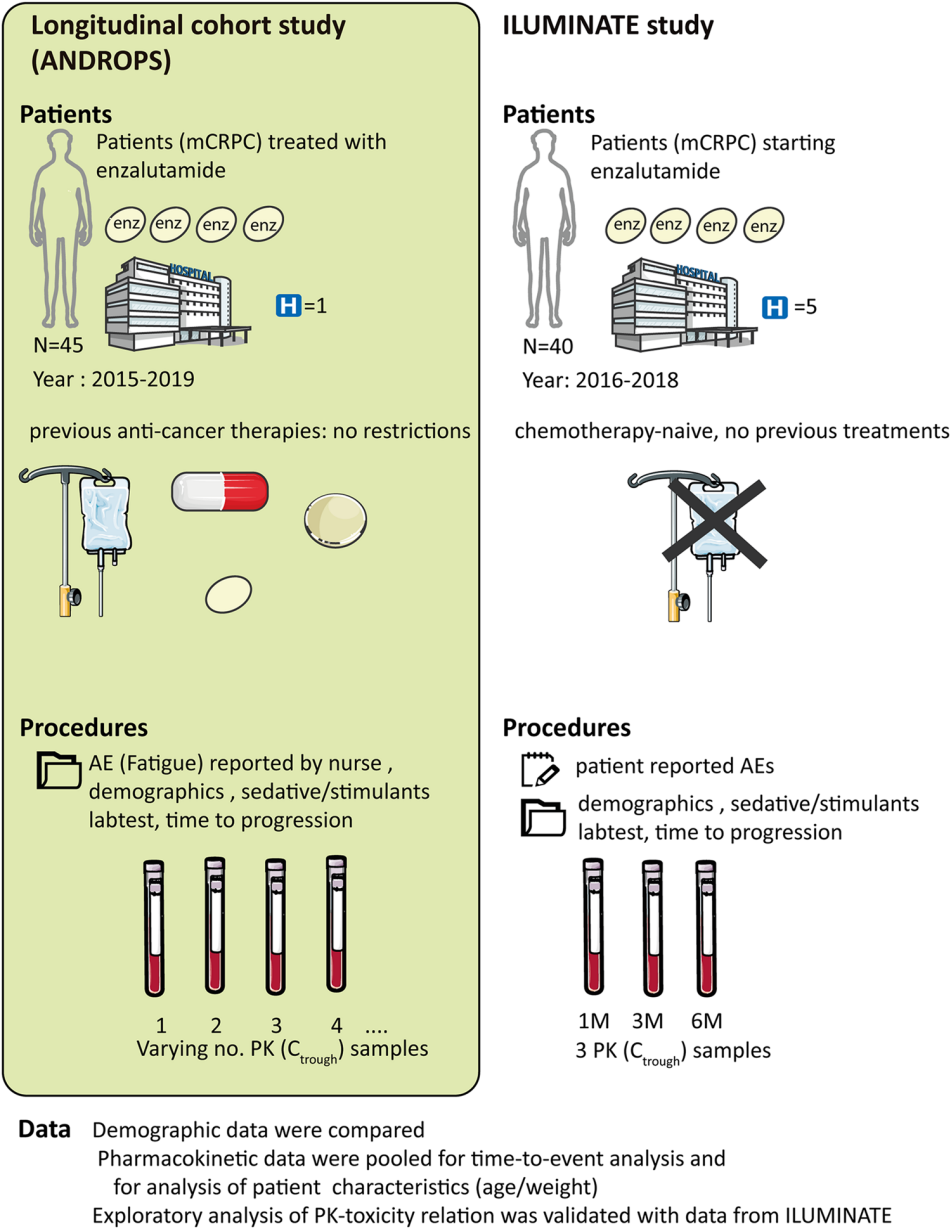
Overview of the ILUMINATE and ANDROPS study

Only pharmacokinetic results at steady state (> 40 days) and during the first 6 months of treatment, at a dose 160 mg, and > 1 h after intake were included for analysis. Fatigue data (yes/no) and data on radiological or biochemical progression were retrospectively collected from the electronic medical record (ANDROPS) or case report form (ILUMINATE). Exclusion criteria for analysis of toxicity were: clinical anemia (Hb < 5.6 mmol/L) and concomitant use of stimulants (e.g. methylphenidate). Both ANDROPS and ILUMINATE were approved by the institutional ethical board. In Fig. 1, an overview of the two studies is shown.

To compare data of both studies, geometric mean levels were compared by independent t test. And to compare time to progression, Kaplan–Meier analysis was performed. When no difference between the two studies was shown, data were pooled for further analysis.

Pharmacokinetic variability within patients was described for the data collected in the ILUMINATE study. To explore the relation of exposure with age (in tertiles) and groups of BMI, geometric mean levels of these groups were compared by analysis of variance (ANOVA).To evaluate the relation of exposure and efficacy, sum of enzalutamide and N-desmethylenzalutamide levels were divided in quartiles and analyzed in relation to time to progression by Kaplan–Meier (KM) curves, and differences between the KM curves were tested for significance by the log-rank test. Finally, to evaluate the prevalence of fatigue in relation to drug exposure, the geometric mean levels of patients with event (fatigue) were compared to the levels of patients without fatigue, by an independent *t* test. PSA response was compared between the two groups (fatigue and no fatigue) to monitor if PSA response was of influence on fatigue.

## Results

Between 2014 and 2019, 46 mCRPC patients were included in the ANDROPS study and 40 patients in ILUMINATE. In six patients, the dose was reduced to 120 or 80 mg enzalutamide based on tolerability (*n* = 3 ANDROPS, *n* = 3 ILUMINATE). For 68 patients (29 ANDROPS/ 39 ILUMINATE) out of the total number (86) of included patients, samples at steady state were available within 6 months from start and results were used for the following analysis. Baseline characteristics for these patients are described in Table 1.

**Table 1:** Patient characteristics at baseline

	ANDROPS (*N* = 29)	ILUMINATE *(N* = 39)	Overall (*N* = 68)
Age (years)	70 (14)	74 (8)	72 (9)
Weight (kg)	87(12)	85 (13)	85 (13)
BMI (kg/m^2^)	26.5(4.5)	28.1(4.5)	26.5 (4.2)
PSA level (ng/mL)	40 (49)	49 (72)	43 (71)
PSA doubling time (months)	2.8 (2.9)	4.1 (4.1)	3.4 (3.3)
Gleason score ≤ 8	55.6%	48.7%	50%
Gleason score > 8	44.4%	51.3%	50%
Hb level (mmol/L)	8 (1.1)	8 (0.9)	8.1 (1)
Albumin (g/L)	36 (4)	41 (6)	38 (5.5)
LDH (U/L)	Not collected	228 (70)	–
WHO Performance score			
0	Not collected	71.8	–
≥ 1	Not collected	28.2	
Chemotherapy (%)	41.4	7.7	22.1 (15)
Previous therapy with abiraterone acetate (%)	6.9 (2)	0	2.9 (2)
Spread of disease (%)			
Bone	31	30.8	29.4
Lymph	24.1	23	22.1
Bone and lymph	37.9	28.2	33.8
Visceral	6.9	17.9	11.8

Continuous values are presented as mean (IQR), Categorical values are presented in *N* (%)

**Fig. 2:**
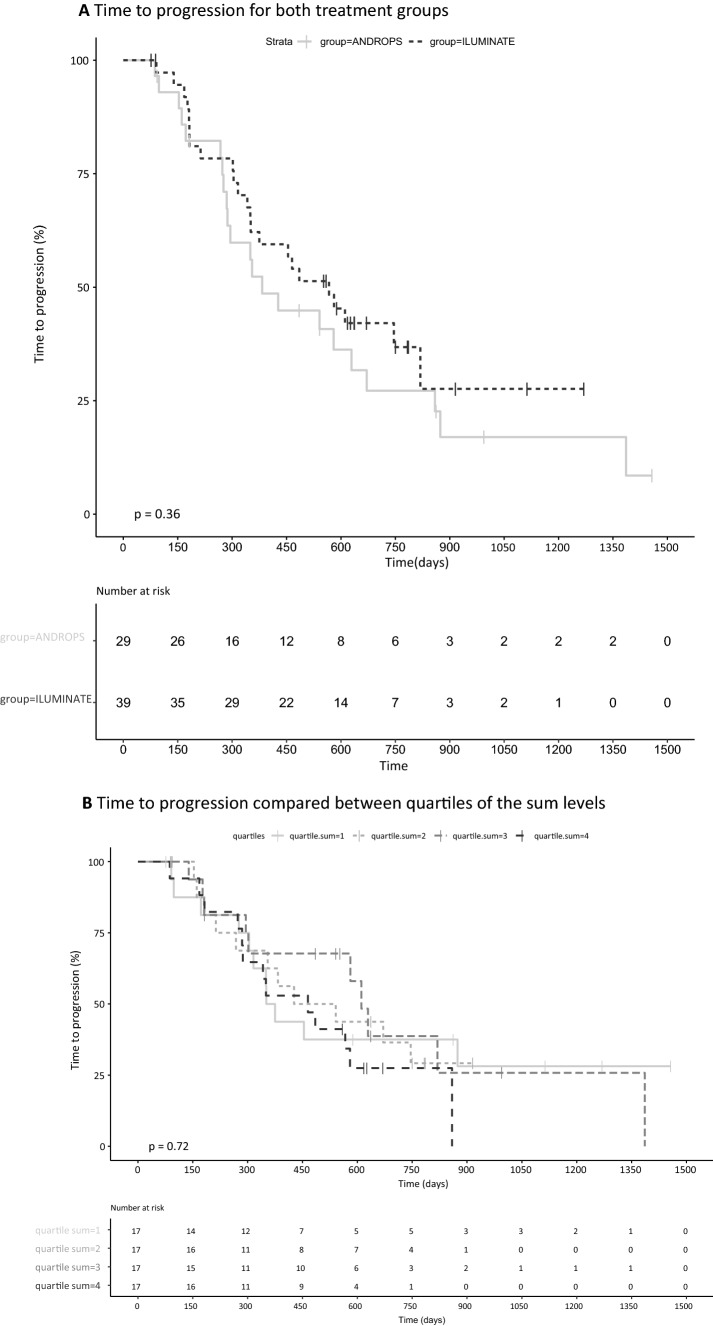
**a** Time to progression for both treatment groups, **b** Time to progression compared between quartiles of sum levels. Quartile 1: 17.6–23.0 mg/L, quartile 2: 23.1–25.4 mg/L, quartile 3: 25.5–29.1 mg/L, quartile 4: 29.6–37.6 mg/L

No statistical difference was observed between geometric mean levels (CV%) of enzalutamide in ANDROPS vs ILUMINATE (12.3 mg/L (20) vs. 13 mg/L (21.2), *p* = 0.26) and N-desmethylenzalutamide (12.9 mg/L (30.1) vs. 13.7 mg/L (23.2), *p* = 0.41). Time to progression was not different between the two studies: in the ANDROPS, study median TTP was 383 days (95% CI 287–859) and in ILUMINATE, the TTP was 567 days (95% CI 351–NR) *p* = 0.36, Fig. 2a. The geometric mean levels (CV%) for the sum (enzalutamide + N-desmethylenzalutamide), enzalutamide alone and N-desmethylenzalutamide alone, were 26.0 mg/L (16.4), 12.6 mg/L (20.6) and 13.6 mg/L (26.1). Within patient variability was 8.8% for enzalutamide and 7.3% for N-desmethylenzalutamide. Geometric mean levels for the sum, enzalutamide and N-desmethylenzalutamide were not different between the different age and BMI groups (Table 2). The median time to progression of the pooled data was 485 days (95% CI 351–671). Time to progression analysis of quartiles of sum levels showed no difference in time to progression between the quartiles of exposure levels (*p* = 0.72), Fig. 2b.

**Table 2:** Enzalutamide and N-desmethylenzalutamide levels related to age and body mass index (BMI)

	Age category: median age (year)				BMI mg/m^2^ (category)			
	I: 61 *N* = 23	II: 73 *N* = 23	III:78 *N* = 22	*P* value	< 25 *N* = 17	25–30 *N* = 36	> 30 *N* = 14	*P* value
Enzalutamide (mg/L); geometric mean (CV%)	12.5 (21.3)	13.2 (21.4)	12.2 (18.8)	0.78	12.2 (20.1)	12.6 (17.9)	13.2 (27.4)	0.31
N-desmethylenzalutamide (mg/L); geometric mean (CV%)	12.5 (25.9)	13.6 (26.9)	14.1 (25.4)	0.15	14.4 (30.4)	13.4 (23.6)	12.4 (25.9)	0.11

One-way ANOVA on geometric mean levels. Analysis performed on pooled data from ANDROPS and ILUMINATE. For one patient BMI was missing (ILUMINATE study)

Five out of 29 patients in the ANDROPS study were excluded for the fatigue analysis: 2 due to clinical anemia for which they were treated, 1 used concomitant methylphenidate, another patient used concomitantly nilutamide, and 1 patient developed an aggressive second primary cancer. Fatigue was observed in 22 patients (36%) (14 ANDROPS + 8 ILUMINATE). For patients with fatigue, the median time to sample collection was 88 days (range 41–176) for patients in the ANDROPS study and in the ILUMINATE study, PK levels and fatigue reports at 3 months from start were included.

No difference in number of patients with fatigue was observed when quartiles of the enzalutamide (*p* = 0.53) and N-desmethylenzalutamide levels (*p* = 0.13) were analyzed by ANOVA. No statistical difference in sum levels, enzalutamide levels alone and N-desmethylenzalutamide levels alone were observed between patients who developed fatigue compared to those who did not develop fatigue (Table 3). The decrease in PSA after 12 weeks was comparable between patients with reported fatigue vs. patients without reported fatigue.

**Table 3:** Patient characteristics and pharmacokinetics vs. occurrence of fatigue

	Fatigue		
	Yes (*n* = 24)	No (*n* = 44)	*P* value
Enzalutamide (mg/L)	12.1 (17.9)	12.9 (21.6)	*0.21*
N-desmethylenzalutamide (mg/L)	13.9 (26.9)	13.2 (25.7)	*0.43*
Sum of enzalutamide and N-desmethylenzalutamide (mg/L)	26.0 (16.4)	26.0 (16.8)	*0.95*
Age (years)	71 (31)	73 (8)	*0.18*
Hb level at time of sampling (mmol/L)	8.2 (1.4)	8 (0.8)	*0.32*
Albumin (g/L)	37 (5)	38 (6)	*0.42*
Previous chemotherapy	7 (29.2)	8 (18.2)	*0.30*
Opioid comedication	4 (16.7)	6 (13.6)	*–*
Sedative comedication^a^	5 (20.8)	2 (4.5)	*–*
Median PSA decrease at 12 wks %	- 82.3 (34)	-77.5 (28)	*–*

Concentration levels are described as mean with CV%, continuous variables as median (IQR), categorical variables as *N* (%)

^a^Benzodiazepines

## Discussion

The aim of this study was to evaluate the influence of age and BMI on enzalutamide and N-desmethylenzalutamide exposure and furthermore, to evaluate the relation between exposure and response and exposure and toxicity (fatigue) in mCRPC patients. No exposure–fatigue relationship, or exposure–response relationship was observed. Both age and BMI did not influence exposure to enzalutamide and N-desmethylenzalutamide. Therefore, we conclude that no dose adjustments are needed based on age or BMI.

Plasma levels in our population were comparable to the PREVAIL data, suggesting comparable exposure in patients outside of clinical trials [19]. Age did not influence the exposure of enzalutamide and N-desmethylenzalutamide which is in line with recently published data by Crombag et al. describing that the exposure of enzalutamide and its metabolites was not influenced by age [13]. Finally, no influence of BMI on the exposure was shown in this study. However, only a small number of patients with high BMI (> 30) were included and, therefore, these results should be treated with caution and cannot be extrapolated to morbid obese patients treated with enzalutamide. In our study population, median age and BMI were comparable to the populations of the prevail and affirm trials [5, 6].

Furthermore, longitudinal exposure was evaluated in a clinical setting and observed very low within patient variability of both enzalutamide and N-desmethylenzalutamide (< 10%) at steady state. This is lower than the data shown in the population pharmacokinetic analysis [15]. This finding might be explained by a lower number of samples per patient in our study in comparison to previous data, although variability observed was also low (< 30%) in the population pharmacokinetic analysis [15]. The low intrasubject variability adds value for individual patients, since a single measurement of plasma concentrations after a dose reduction can provide information on exposure levels during treatment.

Data on fatigue in our study were spontaneously reported by patients (yes/no) and no formal questionnaires such as FACIT-fatigue were used. Also, scoring of fatigue was performed by yes/no answers, therefore, differences in the extent of this side effect may have been missed; which could have led to reporting bias. Furthermore, fatigue is known as a multidimensional symptom and can be influenced by several aspects such as exercising, comorbidities, previous medications and pain. Since these data were not collected, this analysis was not controlled for these effects. However, the prevalence of fatigue in our study (36%) is corresponding to previously published data [3]. Failure of treatment could be of influence on the reporting of fatigue since cancer progression can lead to fatigue. However, in patients with and without fatigue, the decrease in PSA was not different, suggesting that no effect from (biochemical) treatment response on the occurrence of fatigue was suggested. In addition, to reduce potential bias of treatment failure on the reported fatigue, a time frame of the first 6 months since start of treatment was used. The recent AQUARiUS study showed a significant difference from baseline for fatigue level after 3 months of enzalutamide therapy compared to abiraterone, supporting the time frame selected for analysis in our study [20].

No relation between fatigue and the plasma levels of enzalutamide, N-desmethylenzalutamide or the sum, was observed. This may be explained by the hypothesis that with the current dose, both the plateau of the exposure–response curve and the exposure–toxicity curve are reached and no additional toxicity nor efficacy is observed when the exposure increases [19, 21]. Furthermore, due to previously mentioned drawbacks in the evaluation of fatigue, we cannot exclude that patients with severe fatigue are overexposed to enzalutamide. Also, the limited intersubject variability in exposure may hamper the analysis of a pharmacokinetic-pharmacodynamic relation at this dose level. An implication hereof might be that an exposure–toxicity relationship should be evaluated over a broader dose range, including a lower dose, for which a randomized study is currently ongoing (NCT03927391).

Concluding, we investigated the influence of patient characteristics on enzalutamide and N-desmethylenzalutamide levels in mCRPC patients, and observed that age and BMI did not influence systemic exposure. Further structured prospective research is needed to understand the relation between exposure and fatigue, as knowledge on this subject can help clinicians and patients in management of this burdening side effect.
